# The DLPFC is centrally involved in resolving Stroop conflicts, suppressing distracting sensory input within the auditory and visual system

**DOI:** 10.3389/fpsyg.2024.1427455

**Published:** 2024-10-18

**Authors:** Ann-Christine Ehlis, Lisa Zarantonello, Florian B. Haeussinger, Tim Rohe, David Rosenbaum, Andreas J. Fallgatter, Moritz J. Maier

**Affiliations:** ^1^Department of Psychiatry and Psychotherapy, Tuebingen Center for Mental Health, University of Tuebingen, Tuebingen, Germany; ^2^LEAD Graduate School and Research Network, University of Tuebingen, Tuebingen, Germany; ^3^German Center for Mental Health (DZPG), Partner Site Tuebingen, Tuebingen, Germany; ^4^Department of Medicine DIMED, University of Padova, Padova, Italy; ^5^Institute of Psychology, Friedrich-Alexander-Universität Erlangen-Nürnberg, Erlangen, Germany; ^6^Werner Reichardt Centre for Integrative Neuroscience (CIN), University of Tuebingen, Tuebingen, Germany; ^7^Graduate School of Neural and Behavioral Sciences, University of Tuebingen, Tuebingen, Germany; ^8^Fraunhofer IAO, Center for Responsible Research and Innovation CeRRI, Berlin, Germany

**Keywords:** Gratton effect, executive functions, near-infrared spectroscopy, conflict adaptation, Stroop effect, transcranial magnetic stimulation, cognitive control

## Abstract

**Introduction:**

Cognitive control is a prerequisite for successful, goal-oriented behavior. The dorsolateral prefrontal cortex (DLPFC) is assumed to be a key player in applying cognitive control; however, the neural mechanisms by which this process is accomplished are still unclear.

**Methods:**

To further address this question, an audiovisual Stroop task was used, comprising simultaneously presented pictures and spoken names of actors and politicians. Depending on the task block, participants had to indicate whether they saw the face or heard the name of a politician or an actor (visual vs. auditory blocks). In congruent trials, both stimuli (visual and auditory) belonged to the same response category (actor or politician); in incongruent trials, they belonged to different categories. During this task, activity in sensory target regions was measured via functional near-infrared spectroscopy (fNIRS) and electroencephalography (EEG), respectively. Specifically, fNIRS was used to monitor activity levels within the auditory cortex, while the EEG-based event-related potential of the N170 was considered as a marker of FFA (fusiform face area) involvement. Additionally, we assessed the effects of inhibitory theta-burst stimulation—a specific protocol based on repetitive transcranial magnetic stimulation (rTMS)—over the right DLPFC. Non-invasive brain stimulation is one of the few means to draw causal conclusions in human neuroscience. In this case, rTMS was used to temporarily inhibit the right DLPFC as a presumed key player in solving Stroop conflicts in one of two measurement sessions; then, effects were examined on behavioral measures as well as neurophysiological signals reflecting task-related activity in the frontal lobes and sensory cortices.

**Results:**

The results indicate a central role of the DLPFC in the implementation of cognitive control in terms of a suppression of distracting sensory input in both the auditory cortex and visual system (FFA) in high-conflict situations. Behavioral data confirm a reduced Stroop effect following previous incongruent trials (“Gratton effect”) that was only accomplished with an intact DLPFC (i.e., following placebo stimulation).

**Discussion:**

Because non-invasive brain stimulation is uniquely suited to causally test neuroscientific hypotheses in humans, these data give important insights into some of the mechanisms by which the DLPFC establishes conflict resolution across different sensory modalities.

## 1 Introduction

Humans make decisions guided by their internal goals and external environmental demands, the latter of which are largely perceived via sensory processing. Thereby, human behavior is characterized by a remarkable degree of flexibility that is based on “executive functioning” or “cognitive control.” A major component of cognitive control is the resolution of conflict in light of competing response options or information (e.g., Allport, 1987; Durston et al., 2003). Specifically, cognitive control is needed when stimuli priming competing response options are processed simultaneously, thereby causing response conflict. During cognitive tasks, such conflict or interference effects are reflected in increased reaction times (RTs) and/or increased error rates during incompatible trials of the Stroop task where, for example, the word “red” is written in blue ink and word meaning should be ignored to indicate the print color (e.g., Ehlis et al., 2005). Interestingly, subsequent to high-conflict trials, interference-related behavioral deficits are often reduced or even abolished (e.g., Botvinick et al., 2001; Botvinick et al., 1999; Kerns et al., 2004). This so-called conflict-adaptation or “Gratton effect” (Gratton et al., 1992) is assumed to be the result of processing adjustments in cognitive control that reduce the impact of conflicting stimulus–response translations (Botvinick et al., 2004). Neuroanatomically, cognitive control has been linked to prefrontal areas (Miller and Cohen, 2001) and corresponding executive processes (Itthipuripat et al., 2019), specifically the dorsolateral prefrontal cortex (DLPFC; Durston et al., 2003; Gbadeyan et al., 2016; Kerns et al., 2004; MacDonald et al., 2000; Milham et al., 2003; Milham et al., 2001). It has previously been shown that the DLPFC works in concert with medial prefrontal areas to regulate behavior and optimize response outcomes (e.g., Ridderinkhof et al., 2004; Silton et al., 2010). However, the neural mechanisms by which the DLPFC exerts control during conflict resolution are still partly unknown.

To further address this issue, Egner and Hirsch (2005) developed a Stroop task comprising face stimuli of well-known actors and politicians. As a second stimulus dimension, names were written across these pictures that either matched or did not match the category of the face stimuli. Participants were asked to focus on the pictures (“face instruction”) or the written names (“reading instruction”) and indicate the target category (actor/politician). As in most Stroop tasks, congruent and incongruent stimuli were presented. For example, the name “Al Pacino” written across a picture of Jack Nicholson would constitute a congruent stimulus, as both individuals belong to the same response category of “actors.” On the other hand, the name “Bill Clinton” written across the face of Jack Nicholson would be an incongruent stimulus, as these two individuals belong to different categories (one is a politician, the other an actor) and should therefore prime opposing response tendencies. Please note that, in this particular task, “congruency” of both stimulus dimensions was defined in terms of “response congruency.” That means that if both individuals belonged to the same category (i.e., either both actors or both politicians)—and thus required the same response—the stimulus was defined as congruent, even though the name never matched (identity-wise) the picture depicted with it. Using this elegant task design comprising one stimulus category (faces) with a specific neural region of interest (fusiform face area/FFA), the authors found significantly increased activation under conditions of high cognitive control not only in the DLPFC, but also the FFA during the face instruction. The FFA is an extrastriate visual region responsible for face processing (Kanwisher et al., 1997). The control-related increase in FFA activation mirrored behavioral conflict-adaptation effects in an inverse manner, suggesting a context-dependent top-down modulation of sensory areas by the DLPFC during conflict resolution, leading to an amplified processing of task-relevant stimulus features. However, while FFA activity in the face condition of this Stroop task provided a “window” into the perceptual processing of target and distractor dimensions, the second condition (“word reading”) did not allow for a similarly clear-cut monitoring of related cortical activation. Furthermore, the study design was correlational in nature, so no conclusions can be drawn regarding a potentially causal role of the DLPFC in cognitive control.

In the present study, we therefore implemented a modified facial Stroop task in combination with repetitive transcranial magnetic stimulation (rTMS) and functional neuroimaging with two major aims. First, we wanted to directly test the hypothesis of a *causal* role of the DLPFC in implementing cognitive control; this was accomplished by temporarily inhibiting the DLPFC via rTMS in one of two measurement sessions and then testing effects of this intervention on the resolution of the Stroop conflict and underlying neurophysiological markers. Our second major aim was to further elucidate the *mechanisms* underlying cognitive control *across modalities*.

**Figure 1:**
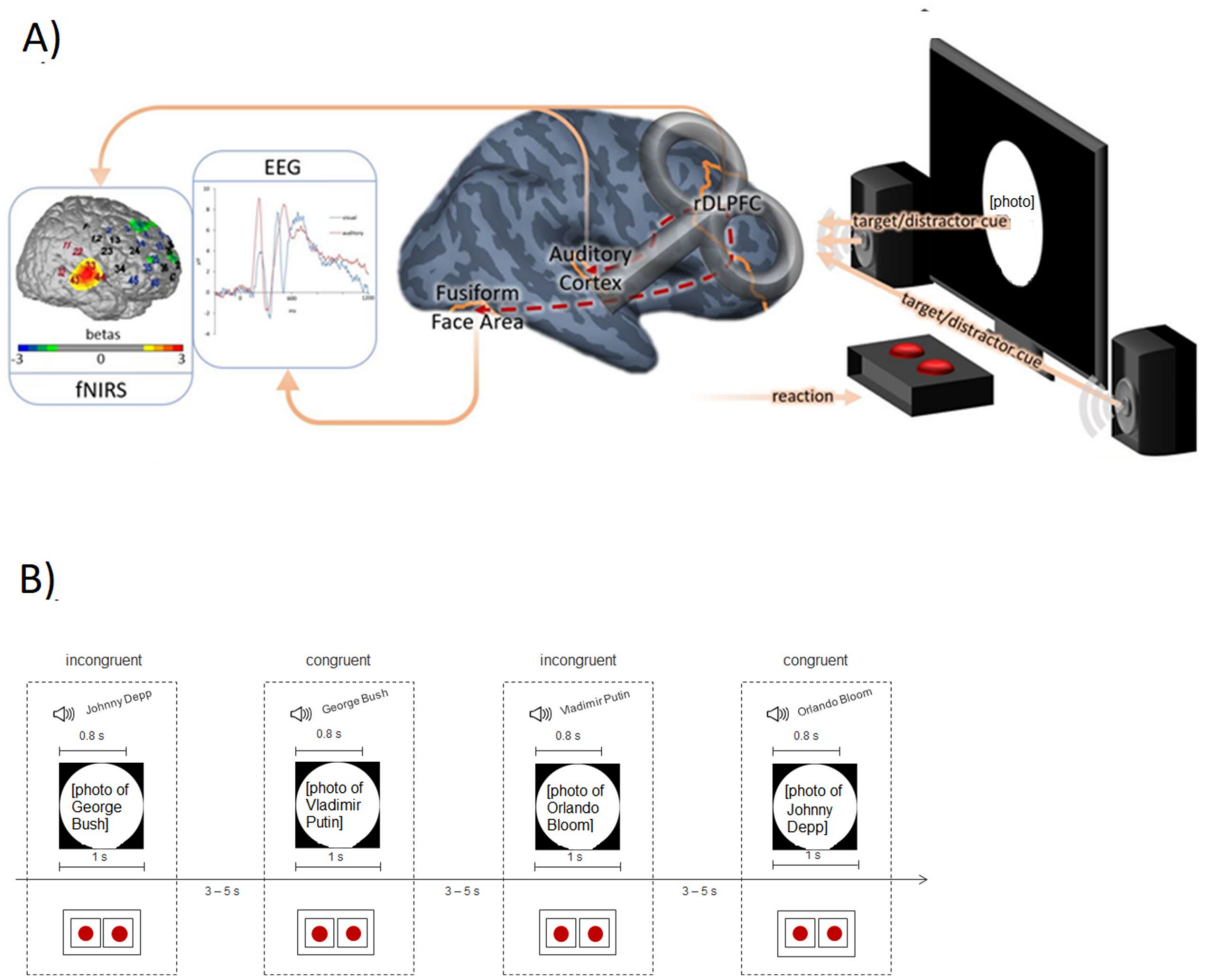
**(A)** Measurement rationale with exemplary data. **fNIRS:** Activation in the auditory cortex (contrast: auditory target condition versus visual target condition) of an exemplary participant. Red channel numbers indicate the candidate channels for the functional channel of interest analysis (see Methods); blue channel numbers are assigned to the DLPFC. **EEG:** Event-related potentials following visual and auditory stimuli at electrode position P7 (grand average; *N* = 25, placebo condition). **Brain:** TMS placebo-verum coil over the right DLPFC (F4 according to the international 10–20 system). The dashed red lines from the DLPFC to the auditory cortex and fusiform face area symbolize the assumed connection between the cognitive control system (DLPFC) and sensory areas. **Experimental setting:** The screen and the speakers emit the target/distractor sensory input depending on the experimental block. Using the red buttons, the participants had to indicate whether they saw/heard an actor or a politician. **(B)** Exemplary trial sequence of the modified Stroop Task (auditory target block) with two incongruent and two congruent trials. The red dots represent the buttons used by the participants to indicate their response (auditory stimulus actor vs. politician).

To this end, we replaced the name reading condition in the above-described Stroop task with a second task instruction (listening to names read via speakers) that has a well-defined neural correlate (i.e., the auditory cortex).^1^ During task performance, functional near-infrared spectroscopy (fNIRS) was used to monitor activation within the DLPFC and auditory cortex, while simultaneous electroencephalography (EEG) measurements were additionally conducted to monitor event-related potential (ERP) indices of face processing (N170; see Figure 1A). This method combination was applied because—while fNIRS is generally better suited than EEG to assess activation changes in circumscribed cortical areas—the FFA is located within the fusiform gyrus and not well accessible with fNIRS which has a limited penetration depth. Therefore, the well-established ERP parameter of the N170 was additionally used to reflect FFA activity in this study. With this design, we tested whether DLPFC activity during the implementation of cognitive control was differentially associated with FFA vs. auditory cortex activity depending on the specific task demands.

This study design extends the scope of previous investigations that also applied inhibitory and/or excitatory neurostimulation during or before (unimodal) Stroop or Flanker tasks to observe subsequent behavioral effects but without concurrent neuroimaging (e.g., Baumert et al., 2020; Friehs et al., 2020; Gbadeyan et al., 2016; Lehr et al., 2019; Li et al., 2017; Parris et al., 2021; Vanderhasselt et al., 2007; Vanderhasselt et al., 2006) (for details on corresponding prior results, please see below in the hypothesis and methods section as well as the discussion). Additionally monitoring task-relevant sensory areas with the described EEG/fNIRS method combination allows for an examination of the *mechanisms* by which the DLPFC exerts cognitive control, in addition to determining a causal role of the DLPFC in these processes. On the other hand, some prior neuroimaging studies have already shed some light on different attentional mechanisms potentially underlying the resolution of Stroop conflicts (Polk et al., 2008; Purmann and Pollmann, 2015). Specifically, for classical Stroop color word tasks, functional magnetic resonance imaging (fMRI) studies revealed mechanisms of feature-based attention, in terms of an enhanced neural processing of attended features (i.e., print color; Polk et al., 2008; Purmann and Pollmann, 2015) and partly also a suppressed processing of stimulus-features that served as distractor (i.e., word meaning; Polk et al., 2008). However, these studies were restricted to unimodal Stroop tasks (i.e., the visual modality) and not explicitly related to DLPFC functioning. So, in summary, the present study design contributes to the literature by combining non-invasive brain stimulation as an experimental tool to probe causality with functional brain imaging and behavioral data during an audiovisual Stroop task, to link specific attentional mechanisms underlying the resolution of Stroop conflicts across stimulus modalities directly to the DLPFC. The described neuroscientific methods were applied in order to elucidate mechanisms underlying observable behavioral effects of conflict resolution.

With our experimental setup, we specifically aimed to test two primary hypotheses:

1. Behavioral data: Following an inhibitory stimulation of the right DLPFC, the Gratton effect (i.e., a reduced Stroop effect on incongruent following incongruent compared to incongruent following congruent trials)—as a behavioral marker of successful implementation of cognitive control in high-conflict situations—should be significantly reduced due to the assumed central role of the DLPFC within the cognitive control network. Previous behavioral studies support this hypothesis by showing either reduced or increased interference sequence/interference expectancy effects (i.e., Gratton effects) following disruptive or excitatory neurostimulation, respectively, over the right DLPFC (Friehs et al., 2020; Gbadeyan et al., 2016; Vanderhasselt et al., 2007).
2. Neurophysiological data: After inhibiting the right DLPFC, conflict-related activity *modulations* within task-relevant sensory areas should be significantly reduced, as we assume these modulations to be initiated by the DLPFC (in response to high-conflict situations, underlying the behavioral Gratton effect). Specifically, conflict-related modulations of auditory cortex activation as well as activity levels in the FFA should be significantly reduced following inhibitory prefrontal stimulation. Please note that activity levels in the auditory cortex were assessed by the fNIRS hemodynamic response, whereas FFA activity was monitored via N170 amplitudes.
3. Going one step back, we also hypothesized—as the methodological basis of our experiment (plausibility checks)—to see (a) overall reduced prefrontal activation following inhibitory brain stimulation (compared to sham stimulation) and (b) increased DLPFC activity for incongruent following incongruent Stroop trials, i.e., when cognitive control should already be established by a preceding high-conflict trial (assumed prefrontal correlate of the Gratton effect).

More exploratory (secondary) hypotheses concern potential correlations between the Gratton effect as an objective marker of conflict-related regulation and questionnaire data reflecting real-life disturbances of cognitive control. The rationale behind this is the following: Attention-deficit/hyperactivity disorder (ADHD) as well as the subclinical trait of impulsivity have been associated with difficulties in cognitive control functions, leading—amongst others—to increased Stroop/interference effects (Lansbergen et al., 2007) and alterations in neurophysiological correlates of conflict-monitoring (Ehlis et al., 2018; Herrmann et al., 2009). Therefore, we additionally included a questionnaire to assess ADHD symptoms in our sample of adult participants, the Adult ADHD Self-report Scale (ASRS; Adler et al., 2006). Furthermore, subjective measures of perceived cognitive failures and attentional/inhibitory control in everyday life were included by means of the Cognitive Failures Questionnaire (Lumb, 1995) and the Adult Temperament Questionnaire (Wiltink et al., 2006), respectively. These questionnaires were applied in order to correlate objective neurocognitive performance with subjective real-life strengths/deficits.

## 2 Materials and methods

### 2.1 Participants

The participants were recruited via an online platform for participants in Tübingen. They first had to complete a short questionnaire to determine the suitability for the experiment. Exclusion criteria were any contraindications for transcranial magnetic stimulation/TMS [cf. Rossi et al. (2009)], any chronic or acute diseases which can influence the cerebral metabolism (e.g., moderate or severe craniocerebral trauma, kidney insufficiency, diabetes, unattended hypertension), neurological or psychiatric diseases (present or past) or acute endangerment of self or others. The implementation of this study was in accordance with the current version of the Declaration of Helsinki. A positive ethics vote was obtained from the ethics committee of the Medical Faculty of the University of Tübingen. Written informed consent was obtained from the participants after detailed information about the study.

A total of thirty-one healthy adults (18 female, 13 male, 0 diverse) aged between 18 and 31 years (*M* = 23.55, *SD* = 3.68) participated in this study which was designed as a cross-over experiment with two measurement time-points for each participant. All of them were college students at the University of Tübingen at the time of data collection and received financial compensation for their invested time. No participant reported any history of psychiatric or neurological disorders and all of them indicated to be right-handed, native German speakers. Power calculations to determine approximate sample size requirements were based on previous rTMS studies of our group (e.g., Tupak et al., 2013). In more detail, Tupak et al. (2013) examined the effects of an inhibitory rTMS challenge on prefrontal oxygenation as well as behavioral data in a group of healthy participants performing an emotional Stroop paradigm combined with fNIRS. However, instead of a cross-over design, classical challenge sessions were conducted (baseline measurement—rTMS intervention—post-rTMS assessment). For right-hemispheric stimulation, the within-subject effect of the rTMS protocol (regarding task-related changes in cerebral oxygenation for the baseline vs. post-rTMS measurement) reached an effect size *f* of 0.40 (based on a partial η^2^ of 0.139 as derived from the repeated-measures ANOVA; data obtained from the first author). Assuming a predefined *p* of 0.05 and a power criterion of at least 90%, this effect size would correspond to a total sample size of *N* = 19. Considering the increased risk of data dropouts in our cross-over design comprising two measurement sessions, we decided to include a few more subjects during recruitment and aimed at a total sample of 30 participants (in order to ensure a final analyzed sample of at least 20 participants with available data for both sessions).

### 2.2 Experimental design

The present study was a within-subjects design with two identical measurement sessions two weeks apart in which participants received either verum or placebo/sham continuous theta-burst stimulation (cTBS). Verum stimulation meant that an inhibitory rTMS protocol was applied over right prefrontal areas, whereas during placebo/sham stimulation, the magnetic pulses were not actually angled toward the brain (and thus no inhibitory stimulation occurred). Between the cTBS and the experimental paradigm, approximately 7 min were needed for fitting the NIRS cap. The order of the cTBS manipulation (verum or sham for the first vs. second measurement time-point) was counterbalanced across participants, and participants as well as the experimenter were blinded with respect to the current cTBS protocol (verum vs. sham). The questionnaires were assessed online before the first measurement session.

### 2.3 Paradigm

The paradigm was a modified facial Stroop interference task, similar to the one used by Egner and Hirsch (2005), with congruent and incongruent Stroop stimuli. However, the stimuli were adapted for use in a German sample (see below) and instead of the “word reading” condition previously employed, we presented interfering auditory cues as the second stimulus dimension/task instruction. More precisely, photographic face stimuli depicting well-known actors and politicians were displayed, while names were heard simultaneously via loudspeakers. For the congruent condition, the auditory cue matched the current face stimulus regarding the stimulus *category* (and thus the correct response). For example, a spoken “George Bush” was combined with a picture of Vladimir Putin, so both stimulus dimensions primed the same response: “politician.” Please note that both stimuli never matched regarding the actual identity of both individuals. For incongruent trials, the auditory cue belonged to the stimulus category currently NOT displayed on the screen. For example, “George Bush” was combined with a picture of the actor Johnny Depp. There was one audio target block and one visual target block; each block consisted of 144 trials and the order of the two blocks was counterbalanced across participants. Furthermore, the order of the trials was balanced for congruency of the current and previous trial with four possible combinations: congruent followed by congruent, congruent followed by incongruent, incongruent followed by congruent, and incongruent followed by incongruent. The visual stimuli were presented for 1 s; the auditory stimuli had a duration of 800 ms. Between the stimuli, there was a jittered break for 3–5 s. Based on the results of a pre-study (see below), we used a stimulus onset asynchrony (SOA) of 0 ms for the auditory target block and an SOA of 150 ms (distractor first) for the visual target block, to create an equally strong Stroop effect. The participants indicated their response (i.e., “actor” versus “politician” with respect to the auditory or visual target) via a millisecond-accurate USB response pad key (Black Box ToolKit®, United Kingdom), with button order counterbalanced across participants. After the response had been indicated, the stimuli disappeared and the duration of the break was appropriately extended. Between the two blocks, a longer break was inserted, the duration of which was determined by the participants. In Figure 1B, an exemplary trial sequence is depicted.

Overall, we employed a four-factorial experimental design with factors “current congruency” (congruent vs. incongruent current trial), “previous congruency” (congruent vs. incongruent preceding trial), “target modality” (auditory vs. visual target) and “TBS protocol” (verum vs. placebo; see below).

### 2.4 Pre-studies

To identify the best-known actors and politicians in Germany, we performed a pre-study with 50 participants (separate from the study sample of the main trial). In a questionnaire, participants indicated the name of the pictured person and judged the actor’s/politician’s popularity and their personal as well as alleged general familiarity with them. Based on these results and additional analyses (of word lengths, number of syllables and similarities), we eventually decided to use George Bush, Vladimir Putin and Joachim Gauck for the politician category and Johnny Depp, Orlando Bloom and Till Schweiger for the actor category. The pictures were transformed into greyscale and controlled for contrast, intensity and size (300 × 450 pixel, presented in the middle of the screen). The auditory files were edited with audacity® (version 2.1.2) and controlled for volume (approximately 80 decibels), length and peaks. As there are partly contradicting results considering the optimal SOA to elicit a Stroop effect (e.g., Donohue et al., 2013; Hanauer and Brooks, 2003; Roelofs, 2005; Vroomen and Keetels, 2010), we titrated the best fitting SOA for our paradigm in an additional pre-study. Based on the results of this pre-study (see Supplementary material), we used an SOA of 0 ms for the auditory target block and an SOA of 150 ms for the visual target block of the main study.

### 2.5 Theta-burst stimulation (TBS)

Neurostimulation of the right DLPFC was performed with theta-burst stimulation (TBS; Huang et al., 2005; Huang and Rothwell, 2004). The TMS coil was located at electrode position F4, corresponding to parts of the DLPFC according to Herwig et al. (2003). The stimulation followed the protocol developed by Huang et al. (2005): 5 Hz theta bursts of three 50 Hz pulses at 80% individual active motor threshold were continuously applied for 40 s (600 pulses in total). Using this protocol, verum stimulation ought to have had an inhibitory effect on underlying cortical areas (i.e., the right DLPFC). The different TBS challenges were applied in a double-blind fashion using an active-passive placebo/verum coil system by MagVenture®. The coil is two-sided and the system gives the instruction to the blinded experimenter which side to use. In the placebo condition, the magnetic pulses are emitted in a direction away from the participant’s head, so the sound is the same, but no stimulation is induced. Additionally, in the placebo condition somatosensory perceptions on the scalp due to TMS pulses are simulated by smooth electrical impulses provided via electrodes.

Regarding aspects of lateralization, please note that—while both the left and right DLPFC have previously been associated with the Stroop task and cognitive control in general and some controversy exists regarding relative contributions of both hemispheres (e.g., Ambrosini and Vallesi, 2017; Hazeltine et al., 2000; Vallesi, 2021; Vanderhasselt et al., 2009)—the study underlying our current paradigm (Egner and Hirsch, 2005) found specifically the right DLPFC to be involved in cognitive control during conflict adaptation, which is why we chose to inhibit only the right DLPFC for the present project. This decision seems bolstered by previous behavioral studies showing no conflict-specific effects of excitatory stimulation of the left DLPFC (Baumert et al., 2020; Parris et al., 2021; Vanderhasselt et al., 2006).

### 2.6 Measurement methods

#### 2.6.1 Functional near-infrared spectroscopy (fNIRS)

**Figure 2:**
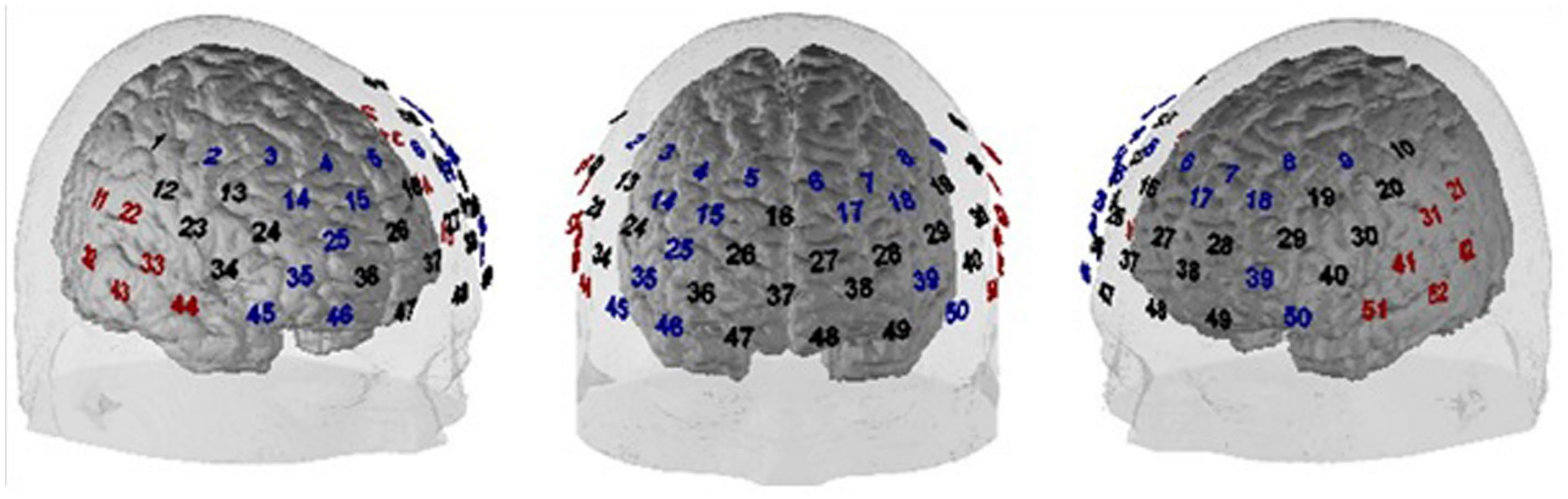
Probe set positioning of the 52 fNIRS channels. Blue channel numbers indicate channels covering the DLPFC, red channel numbers are candidate channels for the functional channel of interest analysis for allocating the individual position of the auditory cortex (for further explanations see section *Preprocessing of fNIRS data*).

FNIRS is a non-invasive optical imaging technique that allows for *in-vivo* measurements of changes in the concentration of oxygenated (O_2_Hb) and deoxygenated (HHb) hemoglobin in cortical brain tissue. FNIRS measurements were conducted using the ETG-4000 Optical Topography System (Hitachi Medical Co., Japan), a continuous wave system with two different wavelengths (695 ± 20 and 830 ± 20 nm) and a temporal resolution of up to 10 Hz. The fNIRS sensors were placed over left and right frontotemporal areas using a 3 × 11 probeset with 52 channels, 16 detectors and 17 emitters, and an inter-optode distance of 3 cm. Based on the international 10–20 system (Jasper, 1958), the bottom row was oriented toward T3/T4 (left/right) and the medial optode in the bottom row was located on Fpz. The probe set positioning is depicted in Figure 2.

#### 2.6.2 Electroencephalography (EEG)

The EEG was recorded from 12 scalp electrodes placed according to the international 10–20 system (Jasper, 1958). Four electrodes were placed at positions P7, P8, O1 and O2 for recording the N170, an early negative ERP component with an occipito-temporal maximum reflecting the processing of face stimuli (e.g., Bentin et al., 1996; Gauthier et al., 2003) within the FFA (e.g., Herrmann et al., 2004; Shibata et al., 2002). One additional electrode was placed on Cz, four around the eyes for correcting eye movements and blinks, one ground between Cz and Fpz and two reference electrodes at the mastoids.

### 2.7 Questionnaires

Different questionnaires were used to assess aspects related to cognitive control such as impulsivity, temperament and cognitive failures in order to investigate potential correlations between Stroop task performance and these individual characteristics. The questionnaires were completed online before (or partly during) the measurement via Sosci Survey (Leiner, 2014). The following instruments were used: Cognitive Failures Questionnaire (Lumb, 1995), Adult ADHD Self-report Scale (ASRS; Adler et al., 2006) and the Adult Temperament Questionnaire (ATQ; Wiltink et al., 2006). For the latter, we focused only on the three subscales of Effortful Control: Attention control, Inhibitory control and Activation control. After the second session, the participants were asked to indicate in which of the two sessions they thought verum cTBS had been applied (to check for blinding).

### 2.8 Data analyses

The behavioral data from a total of 9 participants had to be excluded from the analyses for different reasons: For four participants, RTs were not recorded for at least one of the two sessions (technical error; additionally, one of them also received the wrong Stroop version with a different sequence of target modalities for t1 than for t2); one participant received the wrong version of the paradigm for the second measurement session (i.e., while the assignment of response buttons to stimulus categories [actor/politician] was counterbalanced across participants but kept constant for each individual, one participant had a change in response button assignments between t1 and t2) and two participants confused both response buttons during the “visual” task block of their first measurement session; and finally, two more participants followed the wrong task instruction (visual instead of auditory focus) during the “auditory” task block of their first measurement session.

Three of these participants were also excluded from the analysis of the neurophysiological data, namely participants who actually followed a wrong instruction—i.e. did something that we did not intend to measure—or received an incorrect Stroop version that then confounded a sequence effect with one of the variables of interest. Additionally, seven data sets had to be excluded from the analysis of the fNIRS data due to missing trigger information (*n* = 4), noisy data (*n* = 2) or incomplete recordings (*n* = 1). For the EEG data, a total of three data sets were excluded due to an incorrect positioning of the electrodes (*n* = 1), overall noisy data (*n* = 1) or less than 20 artifact-free segments in at least one of the eight conditions (*n* = 1). Overall, 22 data sets were available for the behavioral analyses (mean age: 23.3 ± 3.8 years; ASRS sum score: 31.7 ± 5.0) while 21 participants could be included in the analysis of the fNIRS (mean age: 23.6 ± 4.1 years; ASRS: 30.9 ± 5.7) and 25 in the analysis of the EEG data (mean age: 23.8 ± 3.9 years; ASRS: 31.7 ± 5.4). So unfortunately, only parts of the sample were available for the different correlation analyses with at least two out of three data sets available (see below; see also limitation section of the discussion).

#### 2.8.1 Preprocessing of fNIRS data

The fNIRS data were exported and analyzed with MATLAB 2020a (The MathWorks, Natick, MA, USA). Preprocessing of the data was performed using customized scripts. After interpolating single noisy channels, spikes primarily caused by movements were removed using Temporal Derivative Distribution Repair (TDDR; Fishburn et al., 2019). After that, O_2_Hb and HHb signals were combined using the correlation based signal improvement procedure (CBSI; Cui et al., 2010) in order to yield a single measure of the BOLD response (“true O_2_Hb”) corrected for remaining motion artefacts (cbsi-Hb). Data were then bandpass-filtered to exclude all frequencies <0.01 Hz and > 0.3 Hz. Event triggers contaminated by biting artefacts or technical noise—as determined by visual inspection of the data—were rejected by excluding affected events (exclusion of ~13% of the events). Finally, a global signal reduction was performed with a spatial Gaussian kernel filter with a standard deviation of *σ* = 50 (Zhang et al., 2016) and data were z-standardized.

**Figure 3:**
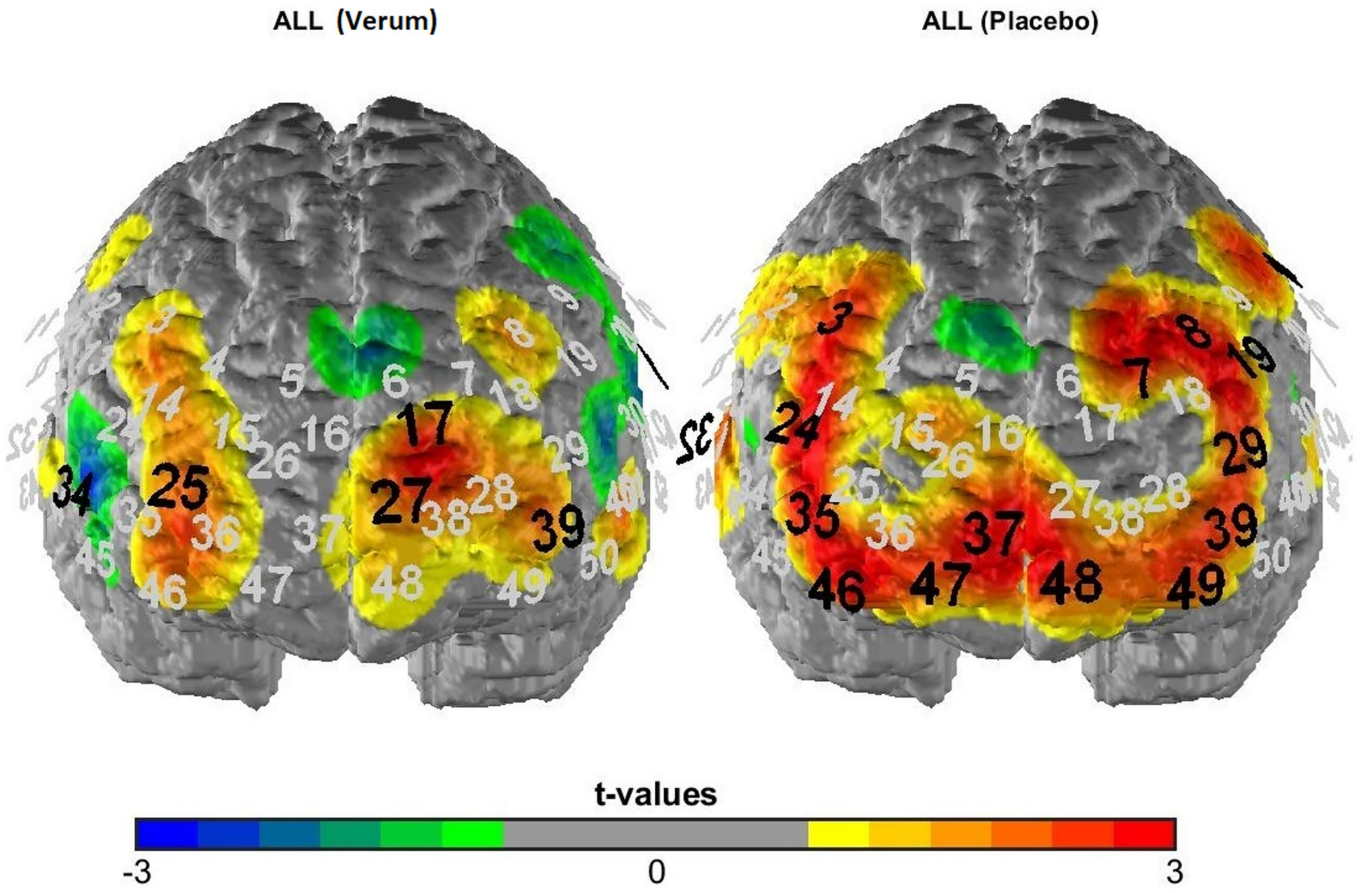
T-maps of fNIRS activation data averaged across all experimental conditions following verum (left) vs. placebo (right) TBS. Black numbers mark significant deviations from zero (uncorrected *p*-values), i.e., significantly activated fNIRS channels.

**Figure 4:**
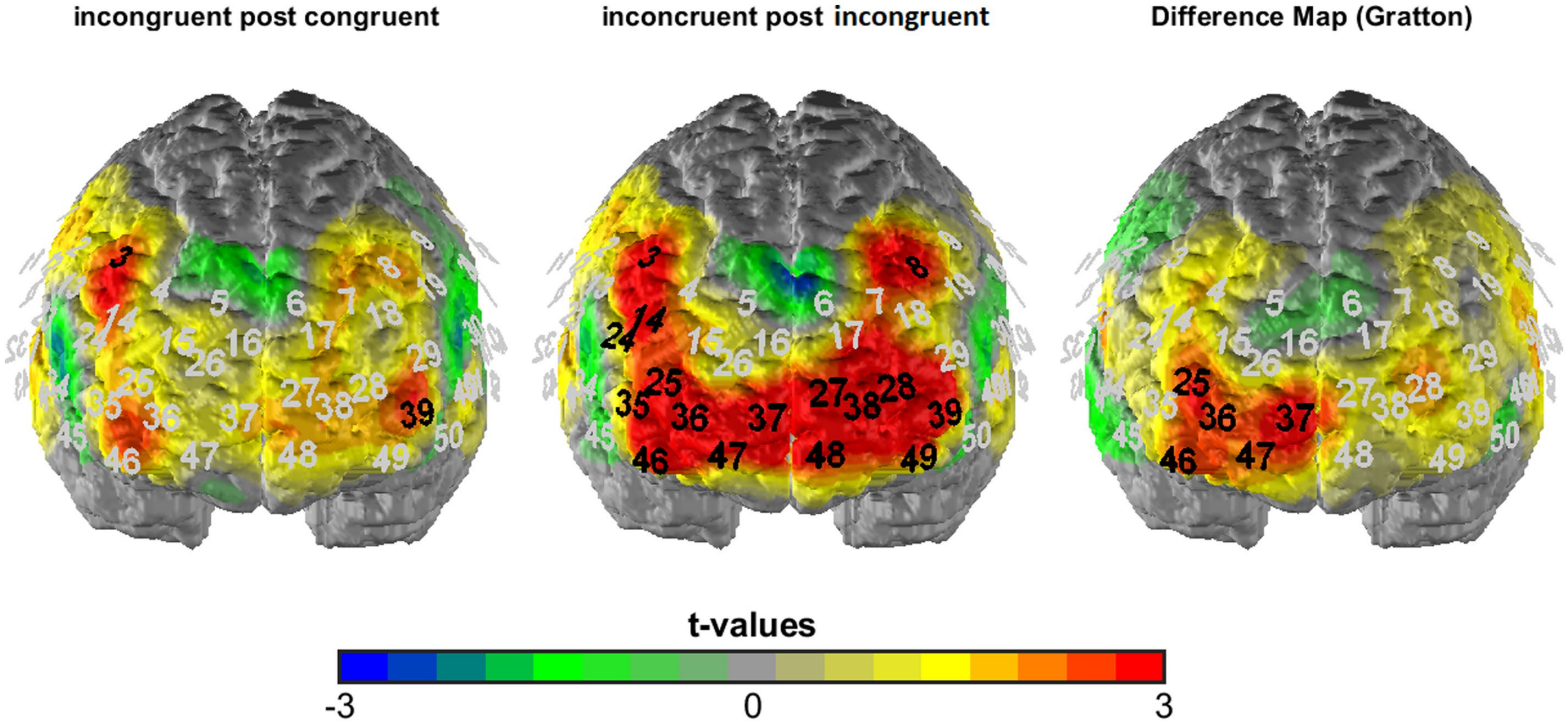
T-maps of fNIRS activation data averaged across visual and auditory task blocks as well as both stimulation conditions (verum and sham) for incongruent following congruent (left) and incongruent following incongruent trials (middle) as well as the corresponding difference map (incongruent following incongruent—incongruent following congruent = Gratton contrast). Black numbers mark significant t-comparisons (against zero: left and middle; between conditions: right; corrected significance level of *p* < 0.01).

Following the pre-processing of the data, we used a model-based analysis in which event-related BOLD responses were modeled by a canonical BOLD response (i.e., one regressor per condition; peak time: 5 s) and which has been adopted for event-related fNIRS data from standard fMRI analyses using the general linear model (GLM) (Plichta et al., 2007). Channels were assigned to approximate underlying cortical areas using the virtual registration procedure described by Tsuzuki et al. (2007), Rorden and Brett (2000) and Singh et al. (2005) (see Figure 2 for an assignment of channels to different regions of interest). Analyses of frontal activation patterns (DLPFC and surrounding channels) were restricted to (1) a plausibility check of our neurostimulation protocol by globally contrasting placebo vs. verum stimulation across task conditions (hypothesis 3a), and (2) a targeted contrast of incongruent following incongruent vs. incongruent following congruent trials to elucidate the cortical correlate of the behavioral Gratton effect (hypothesis 3b). For both of these analyses of frontal cortex effects, simple t-maps were used to illustrate the findings (see Figures 3, 4). We also conducted a ROI (region of interest)-based analysis of the DLPFC to perform correlation analyses (see below). To this end, all channels allocated to the left and right DLPFC, respectively, were averaged per task condition and participant (see Figure 2; left DLPFC: channels #6–9, 17, 18, 39, 50; right DLPFC: channels #2–5, 14, 15, 25, 35, 45, 46). In contrast, to quantify activity within the auditory cortex, we used an individual functional channel of interest (fCOI) analysis since the assignment between temporal channels and underlying auditory cortex is less well defined and signal-to-noise ratio tends to be reduced compared to frontal areas of the brain. To this end, in each participant, we determined the channel with the strongest activation across all conditions (i.e., largest GLM regression coefficient *β* for an all-greater-baseline contrast) from a set of twelve (six per hemisphere) predefined candidate channels (channels: 11, 21, 22, 31–33, 41–44, 51, 52; see Figure 2). This channel was then determined as individual fCOI for the auditory cortex and was used for all subsequent analyses with different subsamples of trials [cf. Powell et al. (2018)]. Importantly, the channel selection contrast (i.e., all greater baseline) was independent from the later contrasts used in the main analyses, so that the procedure avoids circular statistical analysis. This was confirmed in a simulation in which individually selecting a channel based on this selection contrast did not inflate the false positive rate of an independent contrast at group level. The statistical tests were performed on the GLM’s regression coefficient ß using SPSS 28.0 (SPSS Inc., Chicago, United States).

#### 2.8.2 Preprocessing of EEG data

The data analysis was adapted from Herrmann et al. (2004). A 0.1–70 Hz bandpass filter was used and the data were corrected for blinks and eye movements (for the exact procedure see Herrmann et al., 2004). All artifact-free trials (following an automatic artifact rejection excluding segments with amplitudes exceeding ±70 μV or voltage steps exceeding 70 μV from one sampling point to the next) were segmented (−200–1,000 ms) and averaged (in relation to Cz) separately for each subject and condition. Based on these grand mean curves, the N170 was determined as the negative peak value (see Herrmann et al., 2004) in the time frame between 148 ms and 281 ms for electrodes P7 and P8 (correspondingly, the latency of the N170 was individually determined as the time point of the most negative peak within this time-window, separately for both electrode positions). An automatic peak detection was performed, which was visually inspected for all conditions and corrected in single cases where the N170 was not correctly identified by the algorithm. Participants were only further analyzed if they had at least 20 artifact-free segments per task condition.

#### 2.8.3 Statistics

For all analyses of the behavioral data, we used the inverse efficiency score (IES; Bruyer and Brysbaert, 2011; Townsend and Ashby, 1983) which normalizes RTs by the proportion of correct responses: $IES=\frac{\mathrm{reaction}\;\mathrm{times}}{1-\mathrm{proportion}\;\mathrm{of}\;\mathrm{errors}}\text{.}$ A 2 × 2 × 2 × 2 ANOVA for repeated measurements with the within-subject factors stimulation, modality, congruency and congruency of the previous trial was run (only one test, so no correction for multiple statistical testing was deemed necessary for the behavioral data). For post-hoc analyses of significant interactions, paired t-tests were applied as appropriate (with a focus on neurostimulation effects on the Gratton and Stroop effect, in line with our *a priori* hypothesis 1).

To analyze the fNIRS data, we first of all performed a manipulation check of the TBS intervention by analyzing overall activation for the placebo vs. verum stimulation session (averaged across all experimental conditions; hypothesis 3a). The results were depicted in terms of t-maps of the “all > baseline” contrast for the two sessions (see Figure 3) and statistically analyzed using McNemar’s test [comparing the number of significantly activated NIRS channels following placebo vs. verum stimulation (positive *β*-values only)]. After that, in line with the focus of the study, we investigated the cortical correlates of the Gratton effect by contrasting fNIRS activation patterns within the frontal lobe for incongruent following congruent trials vs. incongruent following incongruent trials (hypothesis 3b), again using simple t-maps (see Figure 4). Here, one-sided testing was applied (as we had a clear *a priori* hypothesis of stronger prefrontal activation for previous incongruent as compared to previous congruent trials, reflecting an increased involvement of prefrontal control areas following incongruent Stroop trials, i.e., in “high conflict–high control” conditions), but with a more conservative significance level of *p* < 0.01 (to avoid inflation of the alpha error due to multiple statistical testing). And finally, with respect to our first main hypothesis concerning task-related sensory activation, activity within the auditory cortex was examined based on the different experimental conditions. To analyze a potentially differential modulation of activity levels within the auditory cortex based on the current task modality, intactness of the PFC and current and previous Stroop condition, a 2 × 2 × 2 × 2 × 2 ANOVA for repeated measurements with the within-subject factors stimulation (verum vs. placebo), target (auditory vs. visual), hemisphere (right vs. left), congruency (congruent vs. incongruent) and congruency of the previous trial (congruent vs. incongruent) was run (hypothesis 2; one test, so no correction for multiple statistical comparisons). For analyzing interaction effects, paired post-hoc t-tests were applied as appropriate. Additionally, we correlated activity within the left and right DLPFC with activity in the left and right auditory cortex for the critical task condition as revealed by the above-reported ANOVA (incongruent following incongruent trials for visual task blocks only; exploratory analysis). Here, partial correlations were performed using mean activation across all channels as the control variable, because fNIRS channels (especially neighboring ones) are usually highly dependent and generally positively correlated due to common sources of physiological variance.

With respect to the EEG data and our second main hypothesis regarding a potential modulation of task-relevant sensory (in this case: visual) areas by the PFC (hypothesis 2), we analyzed both latencies and amplitudes of the target ERP (N170). For statistical analysis, a 2 × 2 × 2 × 2 × 2 ANOVA for repeated measurements with the within-subject factors stimulation (verum vs. placebo), target (auditory vs. visual), hemisphere (P7 vs. P8), congruency (congruent vs. incongruent) and congruency of the previous trial (congruent vs. incongruent) was run. As two outcome measures were tested in the electrophysiological domain, we corrected the significance threshold for the two ANOVAs to *p* < 0.025 (Bonferroni correction). Two-factorial ANOVAs and paired t-tests contrasting task and stimulation conditions as well as corresponding difference measures were run for post-hoc analyses (see results section). Additionally, N170 amplitudes and latencies of the most critical task conditions (as revealed by the above-stated ANOVAs) were correlated with corresponding DLPFC activation measures (fNIRS), separately for the left and right DLPFC and both stimulation conditions (placebo, verum) using Pearson’s correlation coefficient (exploratory analyses).

With respect to potential correlations between questionnaire data related to cognitive control and results of the Stroop paradigm (exploratory analysis), we correlated only Gratton difference measures (e.g., IES for visual task blocks of the placebo session: incongruent following congruent minus incongruent following incongruent trials) with the three ATQ scales (Attention control, Inhibitory control, Activation control), the sum score of the Cognitive Failures Questionnaire as well as the ASRS using Spearman correlations (because questionnaire scores were not consistently normally distributed in all of our subsamples [behavioral, fNIRS, EEG] according to Kolmogorov–Smirnov test statistics). In order to avoid alpha error inflation, only significant results with *p* < 0.001 were considered.

For all ANOVAs, partial eta squared was reported for effect size estimation, whereas Cohen’s *d* was used as an effect size for t-test findings. If not explicitly stated otherwise, two-sided testing was applied throughout.

## 3 Results

### 3.1 Behavioral data (hypothesis 1)

**Figure 5:**
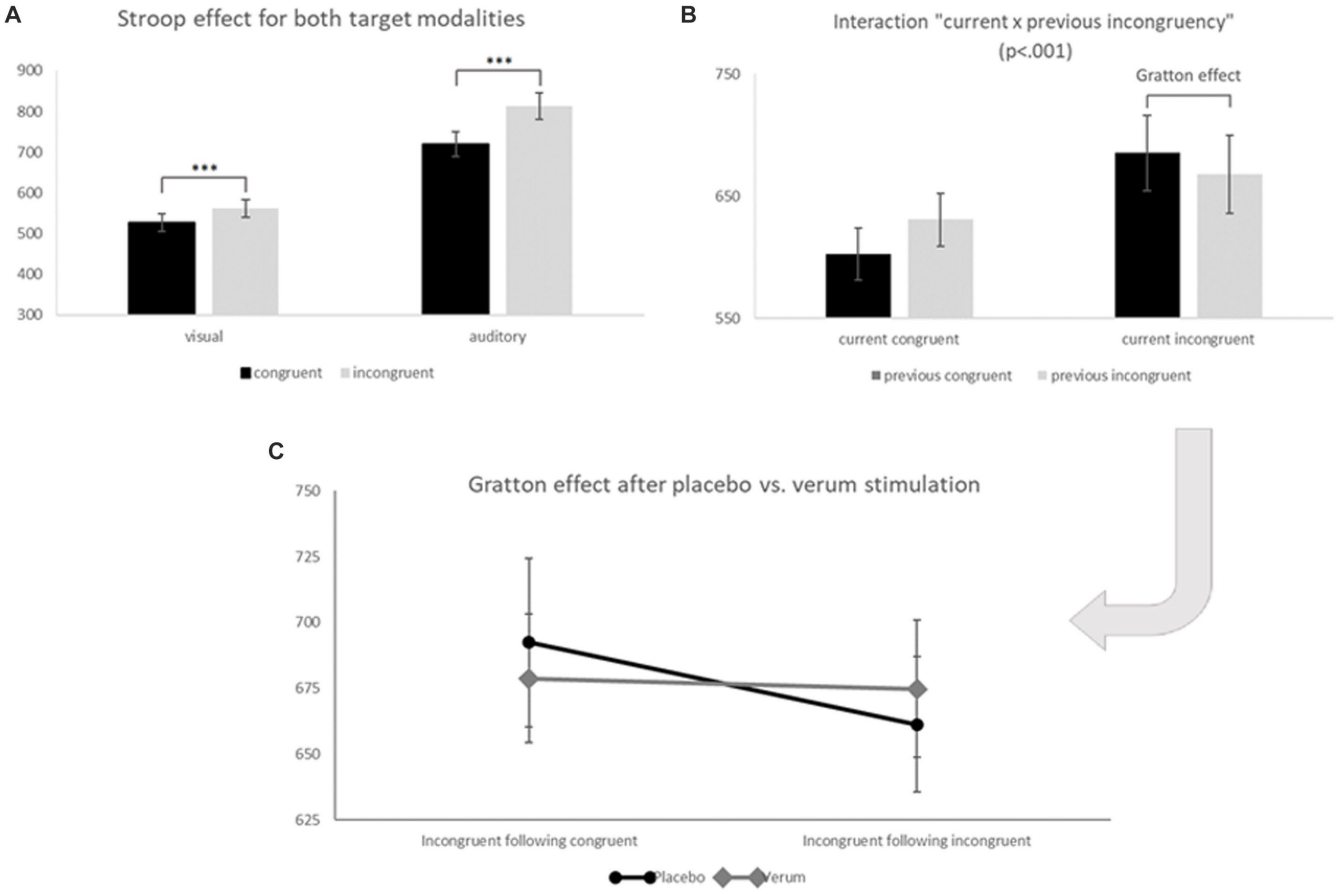
**(A)** Inverse efficiency scores (IES) for congruent (black) and incongruent trials (grey) of visual (left) and auditory (right) task blocks. Significant differences (*** *p* < 0.001) between congruent and incongruent trials show the well-known Stroop effect. **(B)** Inverse efficiency scores (IES) for current congruent (left) and current incongruent trials (right) following congruent (black) or incongruent (grey) previous trials. The comparison within current incongruent trials marks the so-called Gratton effect (IES for previous incongruent < previous congruent trials; *p* < 0.05). **(C)** Inverse efficiency scores (IES) for the TBS placebo/sham (black, round endings) and TBS verum condition (grey, diamond endings) for incongruent following congruent (left) and incongruent following incongruent (right) trials (both conditions significantly differed only for placebo stimulation: *t*_21_ = 2.71, *p* < 0.01 one-sided, 0.05 two-sided = “Gratton effect”). Error bars indicate the standard error (SE).

As expected, we found a strong effect of modality (*F*_1, 21_ = 278.07, *p* < 0.001, η_p_^2^ = 0.930) with a higher IES in the more difficult auditory target condition (*M* = 763.11, *SD* = 143.03) than in the easier visual target condition (*M* = 542.88, *SD* = 98.97). We also found a classical Stroop effect with lower IES for congruent (*M* = 620.81, *SD* = 118.55) as compared to incongruent trials (*M* = 682.34, *SD* = 121.69; main effect “congruency of the current trial”: *F*_1, 21_ = 40.929, *p* < 0.001, η_p_^2^ = 0.661); furthermore, this Stroop effect (i.e., the difference between incongruent–congruent trials) was significantly stronger for the more difficult auditory (*M* = 93.37, *SD* = 76.33) as compared to the visual target modality (*M* = 34.59, *SD* = 33.79; interaction “modality × congruency of the current trial”: *F*_1, 21_ = 12.114, *p* < 0.01, η_p_^2^ = 0.366; see Figure 5A), while it was not significantly modulated by the stimulation condition (non-significant interactions of current congruency and stimulation condition: *F*_1, 21_ = 0.353, *p* = 0.559, η_p_^2^ = 0.017, as well as current congruency, modality and stimulation: *F*_1, 21_ = 1.392, *p* = 0.251, η_p_^2^ = 0.062). A significant two-way interaction “congruency of the current × previous trial” (*F*_1, 21_ = 19.042, *p* < 0.001, η_p_^2^ = 0.476) furthermore revealed the expected Gratton effect, with lower IES for incongruent after incongruent (*M* = 668.03, *SD* = 118.06) as compared to incongruent after congruent trials (*M* = 685.54, *SD* = 124.59) (i.e., lower IES for “high conflict–high control” as compared to “high conflict–low control” trials; *t*_21_ = 2.34, *p* < 0.05; see Figure 5B)^2^. And finally, this effect was additionally modulated by the stimulation condition in accordance with our hypothesis 1 (three-way interaction “stimulation condition × congruency of the current trial × congruency of the previous trial”: *F*_1, 21_ = 4.343, *p* < 0.05, η_p_^2^ = 0.171): When additionally considering the factor “stimulation,” the expected “Gratton effect” (IES for incongruent following congruent trials > incongruent following incongruent trials) was statistically significant only following placebo stimulation (i.e., with an “intact” DLPFC; *t*_21_ = 2.71, *p* < 0.01 one-sided, *p* < 0.05 two-sided), but not after inhibitory TBS over the right DLPFC (*t*_21_ = 0.37, n.s.; see Figure 5C). RTs, error rates and the resulting IES of the behavioral sample are presented in Table 1.

**Table 1:** Reaction times, error rates and IES for the different modalities (visual vs. auditory), stimulation (verum vs. placebo) and congruency conditions (current and previous trial), *N* = 22.

Target modality	Condition	Current trial	Previous trial	RT in ms	SD (RT)	Error in %	SD (Error)	IES	SD (IES)
		Incongruent (IC)	IC	727	184	9.1	7.1	795	159
			C	719	197	11.9	8.0	817	211
	**Placebo**								
		Congruent (C)	IC	711	191	4.6	5.5	747	192
			C	670	173	4.5	4.4	703	181
**Auditory**									
		Incongruent (IC)	IC	738	149	10.6	9.3	827	152
			C	702	137	11.6	9.0	793	121
	**Verum**								
		Congruent (C)	IC	689	149	5.1	6.3	726	147
			C	666	122	4.4	6.0	697	120
		Incongruent (IC)	IC	501	92	7.0	5.2	539	97
			C	509	110	11.7	10.4	581	126
	**Placebo**								
		Congruent (C)	IC	503	103	5.0	5.5	530	105
			C	485	104	5.0	4.7	510	103
**Visual**									
		Incongruent (IC)	IC	516	121	5.9	4.6	547	115
			C	517	103	9.9	9.0	577	118
	**Verum**								
		Congruent (C)	IC	514	115	4.8	6.3	540	116
			C	497	101	4.4	4.2	519	97

### 3.2 NIRS data

#### 3.2.1 Confirmation of stimulation effect (manipulation check; hypothesis 3a)

Figure 3 depicts activation patterns—averaged across all experimental conditions—for the verum and placebo stimulation session. Comparing both stimulation conditions using McNemar’s test (for the comparison of frequencies in dependent samples) revealed a significantly higher number of activated NIRS channels (as indicated by significant, positive t-contrasts against zero) following placebo (15 channels showing significant activation) compared to inhibitory TBS (4 channels showing significant activation; *p* = 0.013), i.e., an overall “stimulation effect” (see hypothesis 3a).

#### 3.2.2 DLPFC—correlates of the Gratton effect? (hypothesis 3b)

In order to investigate the cortical correlates of the overall Gratton effect (reduced Stroop effect following previous incongruent trials), we contrasted fNIRS activation patterns for incongruent trials following incongruent trials vs. incongruent trials following congruent trials. In line with our hypothesis 3b, we observed stronger activation in prefrontal areas on incongruent following incongruent as compared to incongruent following congruent trials, i.e., in our “high conflict–high control” compared to our “high conflict–low control” condition (see Figure 4). While extended prefrontal areas (and only prefrontal areas!) were significantly activated during incongruent following incongruent trials (7 channels over the DLPFC, one channel over the inferior frontal gyrus (IFG), 8 channels over the orbitofrontal cortex [OFC]/frontopolar areas, 2.626 ≤ *t*_20_ ≤ 4.355, *p* < 0.01 [one-sided testing]), the direct comparison between both conditions revealed a cluster of channels related to the Gratton effect that was located within the right prefrontal cortex. Specifically, at a corrected significance level of *p* < 0.01 (one-sided testing), they were localized to Brodman area 46 of the right DLPFC (channels #25 and 46; *t*_20_ = 2.865 and 2.743, respectively; *p* < 0.01), extending into the right frontopolar/orbitofrontal cortex (channels #36, 37 and 47; all *t*_20_ ≥ 2.519, *p* ≤ 0.01).

#### 3.2.3 Auditory cortex (hypothesis 2)

**Figure 6:**
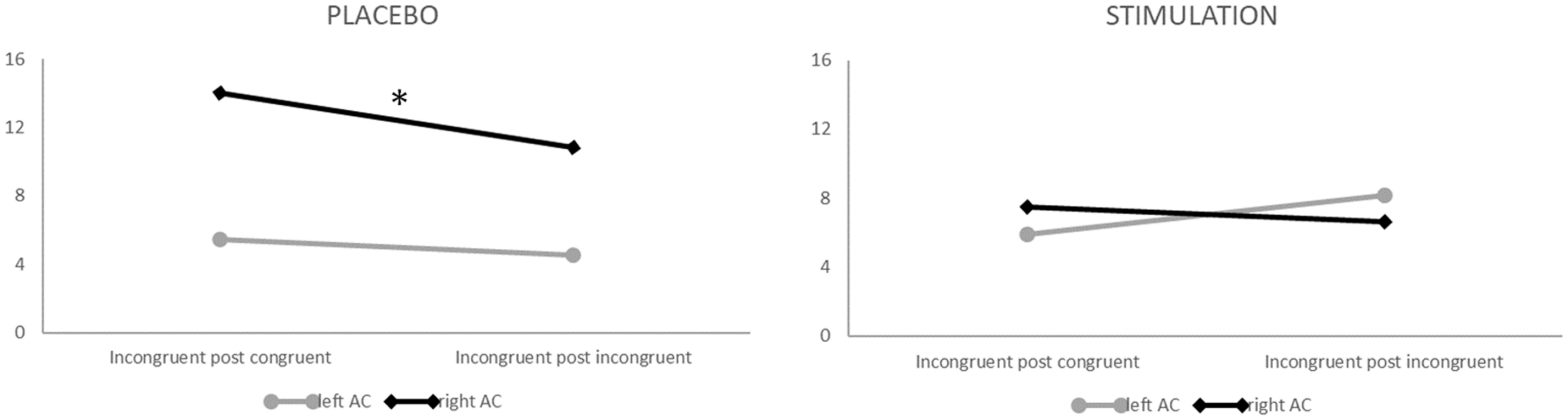
*ß*-values in the left (grey) and right (black) auditory cortex during visual task blocks for incongruent following congruent (left parts of both panels) and incongruent following incongruent trials (right parts of both panels) following placebo (left panel) vs. verum (right panel) TBS. Note that only after placebo stimulation, auditory cortex activity within the right hemisphere was suppressed following previous incongruent trials (i.e., in high conflict–high control conditions; * *p* < 0.05).

For auditory cortex activation, the analyses revealed an interaction effect of stimulation, target, hemisphere, congruency and congruency of the previous trial (*F*_1, 20_ = 4.846, *p* < 0.05, η_p_^2^ = 0.195). Post-hoc analyses of this interaction revealed that—specifically during incongruent trials of visual task blocks, i.e., when auditory input served as the Stroop distractor—right-hemispheric auditory activity was significantly reduced after previous incongruent as compared to previous congruent trials (i.e., in high conflict–high control conditions; *t*_20_ = 1.767, *p* < 0.05), but only after placebo stimulation. This finding indicates a downregulation of distracting auditory input in conflictual (i.e., incongruent) trials when cognitive control should already be established due to a previously incongruent trial (i.e., Gratton effect). Interestingly, this mechanism occurred only with an intact DLPFC but not after inhibitory TBS, which is in line with hypothesis 2. The interaction effect is depicted in Figure 6.

Based on this finding, we were also interested in potential direct correlations between activity within the left and/or right DLPFC and left and/or right auditory cortex during this critical task condition (visual task block, incongruent after incongruent trials). An exploratory partial correlation analysis (control variable: mean activation across all channels in the same condition) revealed a significant negative linear relation between activity within the left DLPFC and right auditory cortex in the verum stimulation session (*r* = −0.493, *p* = 0.027), confirming a potential inhibitory impact of prefrontal control areas on the auditory system in a high-conflict condition where auditory input served as a distractor. However, with a total of eight exploratory correlations, this effect only reached statistical significance at an uncorrected alpha level.

### 3.3 EEG data (hypothesis 2)

#### 3.3.1 N170 latencies

Analysis of N170 latencies only revealed significant main effects of modality (*F*_1, 24_ = 15.320, *p* < 0.001, η_p_^2^ = 0.390) and position (*F*_1, 24_ = 10.457, *p* < 0.01, η_p_^2^ = 0.303), with longer latencies for visual than for auditory task blocks and for the left (electrode position P7) compared to the right side (P8). At a corrected significance level of *p* < 0.025, no other main effects or interactions reached statistical significance (all *F* < 5.7, *p* > 0.025, η_p_^2^ ≤ 0.189).

#### 3.3.2 N170 amplitudes

**Figure 7:**
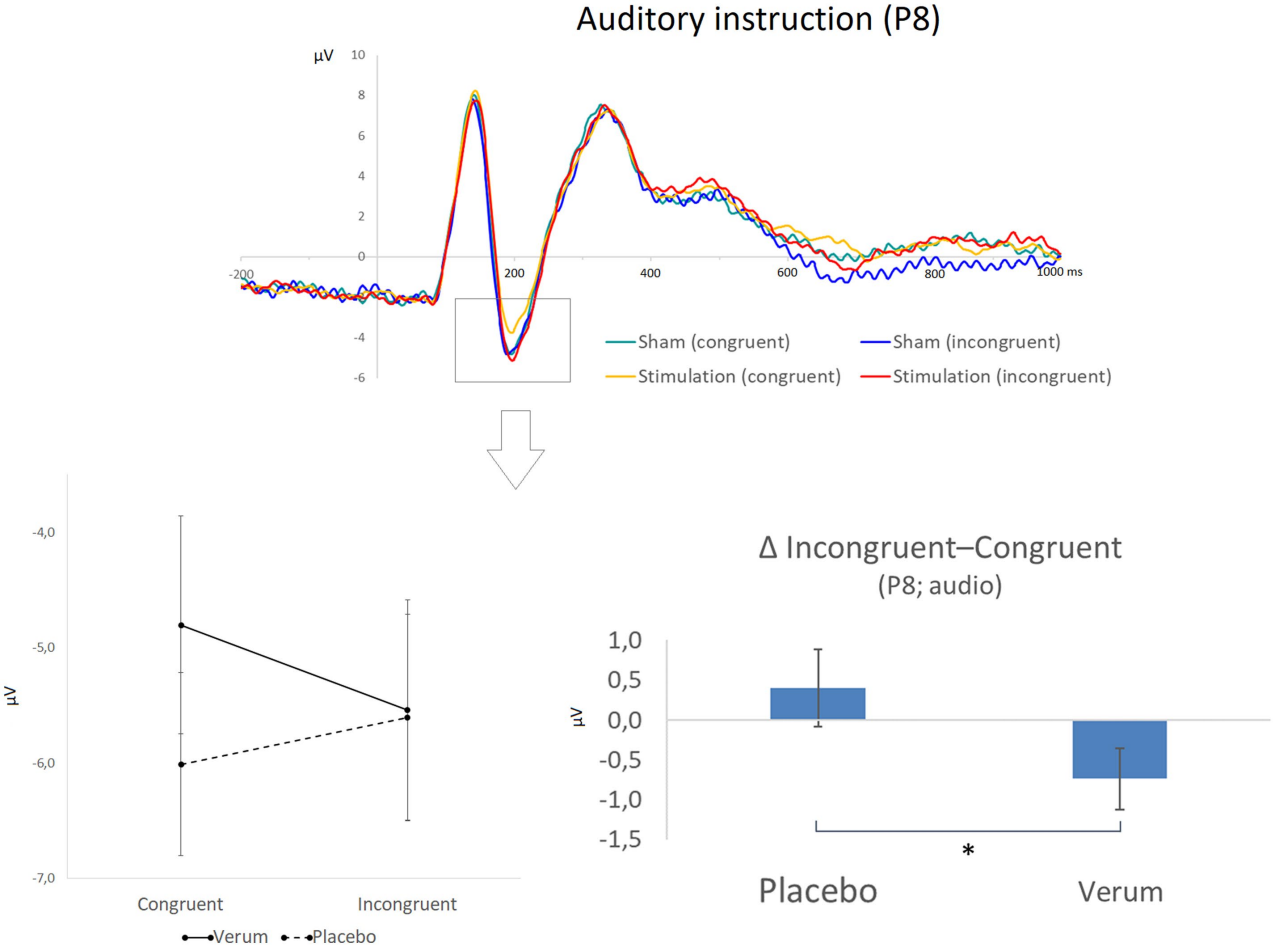
The **top part** of the figure shows grand averaged waveforms for electrode position P8 and a focus on the auditory stimuli (while faces were still depicted), for which significant effects were observed. Different conditions are displayed in different colors (green: congruent stimuli following placebo/sham stimulation; blue: incongruent stimuli following placebo/sham stimulation; yellow: congruent stimuli following inhibitory TBS; red: incongruent stimuli following inhibitory TBS). The N170 peak is highlighted by a box. The **lower part** of the figure illustrates the statistical finding of a reduction of N170 amplitudes (μV) from congruent to incongruent trials only after placebo stimulation (left side; please note that the N170 is a negative potential, so more positive values represent reduced amplitudes). The panel on the right illustrates the significant difference (* *p* < 0.05) between placebo and verum stimulation for the resulting difference measure (N170 amplitude for incongruent minus congruent trials). Error bars indicate the standard error (SE).

For the amplitudes of the N170, a significant main effect of target modality (*F*_1, 24_ = 7.647, *p* < 0.025, η_p_^2^ = 0.242) was observed in addition to an interaction of hemisphere, modality, congruency, and stimulation (*F*_1, 24_ = 5.941, *p* < 0.025, η_p_^2^ = 0.198). Further post-hoc analyses of the interaction effect revealed that—only for *position P8 (right side) and auditory* trials (i.e., when the face stimulus served as a distractor)—a significant interaction effect of current congruency and stimulation condition emerged (*F*_1, 24_ = 4.453, *p* = 0.045, η_p_^2^ = 0.157). This interaction was due to a significantly stronger reduction of N170 amplitudes from congruent to incongruent trials following sham (*Δ* incongruent–congruent: *M* = 0.40, *SD* = 2.40) than following verum stimulation (*M* = -0.73, *SD* = 1.92; direct comparison of Δ between verum and sham: *t*_24_ = 2.110, *p* < 0.05, *d* = 0.422; see Figure 7). Please note that the N170 is a *negative* ERP, so positive values of the difference measure reflect a *decrease* in amplitudes from congruent to incongruent trials. In other words, N170 amplitudes changed differently from congruent to incongruent trials following both stimulation protocols, with a reduced processing of distracting visual input on incongruent compared to congruent trials with an intact DLPFC. This measure was, however, not significantly correlated with DLPFC activity (also Δ of incongruent—congruent trials) as revealed by fNIRS (neither for the left nor right DLPFC and neither following sham nor verum stimulation; all │*r*│ ≤ 0.424, *p* > 0.07).

### 3.4 Correlations with questionnaire data

**Figure 8:**
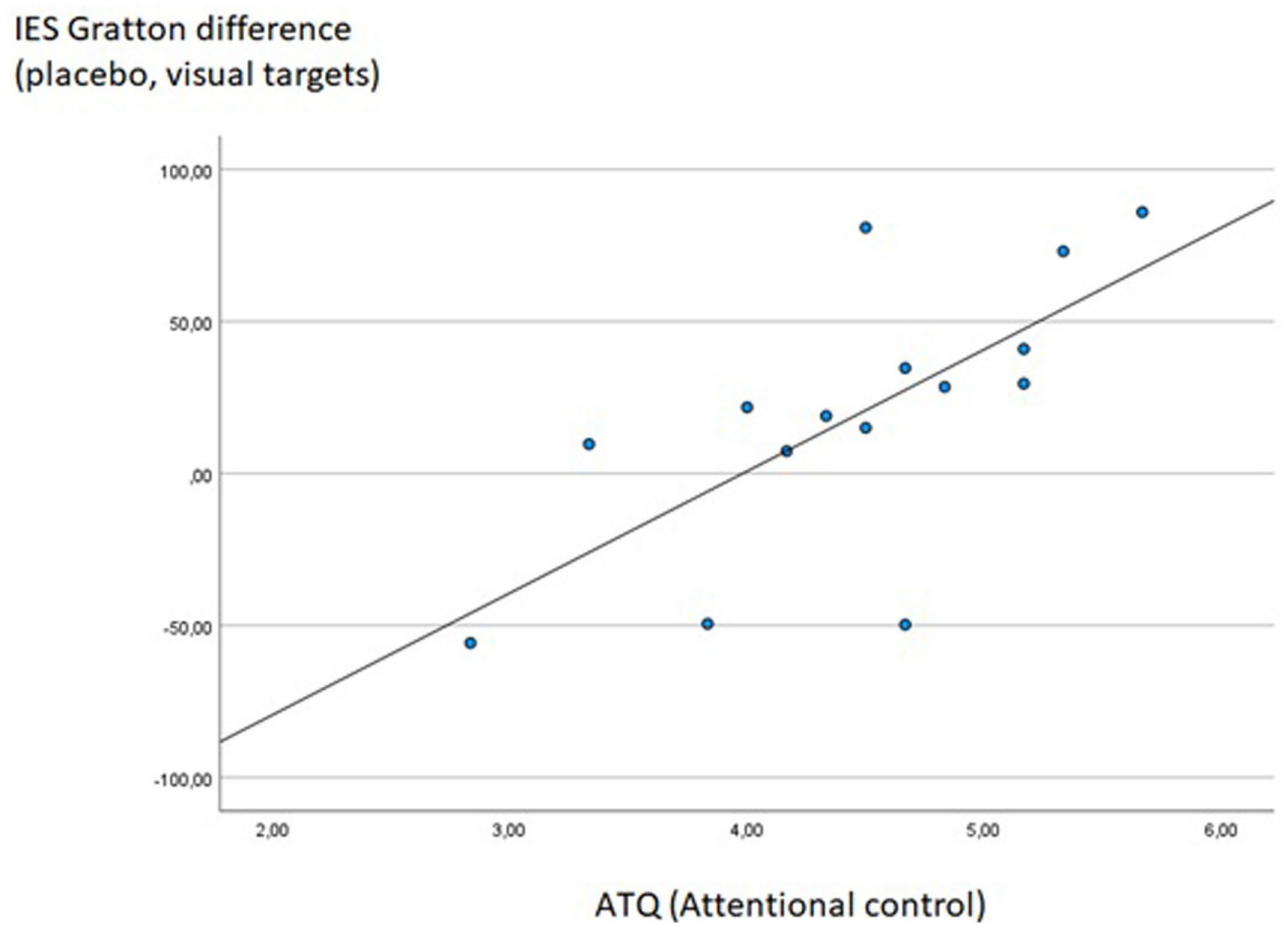
Significant Spearman correlation between ATQ Attention control scores and the IES Gratton difference measure during visual task blocks of the placebo session (IES for incongruent following congruent minus incongruent following incongruent trials); *Rho* = 0.747, *p* < 0.001.

The score of the ATQ subscale “Attentional control” was significantly correlated with the Gratton effect of the IES (difference between incongruent following congruent minus incongruent following incongruent trials) in visual task blocks of the placebo session (*Rho* = 0.747, *p* < 0.001), i.e., the higher participants scored on attentional control, the stronger the reduction of the Stroop effect following previous incongruent trials (see Figure 8). Interestingly, at an uncorrected significance level, this behavioral effect was accompanied by a significant correlation between attentional control scores and activity of the right DLPFC in the same condition contrast (i.e., difference between incongruent following incongruent minus incongruent following congruent trials for visual task blocks of the placebo session; *Rho* = 0.516, *p* = 0.017), indicating that the positive linear relation between attentional control at a self-report level and cognitive control in terms of the Gratton effect may have been mediated by activation levels in the right DLPFC. Only one other correlation between questionnaire data and Stroop measures reached statistical significance at a *p* < 0.001 threshold: The sum score of the Cognitive Failures Questionnaire correlated significantly with the “Gratton difference” within the right DLPFC, again during visual task blocks of the placebo session (*Rho* = −0.686, *p* < 0.001), indicating a reduced increase in DLPFC activation in the “high conflict–high control” condition in participants scoring high on a self-report measure of cognitive failures.

## 4 Discussion

In this study, a multisensory Stroop task was used to further investigate mechanisms implemented by the DLPFC to exert cognitive control over sensory areas in high-conflict situations. Following both verum and placebo TBS, a classical Stroop effect was detected, with significantly higher IES for incongruent as compared to congruent stimuli. Moreover, there was a main effect of the target modality (visual vs. auditory) which is, however, not interpretable due to the different SOAs. *After previous incongruent* trials, the Stroop effect was significantly reduced (“Gratton effect”; Gratton et al., 1992), indicating a more efficient resolution of the Stroop conflict when cognitive control areas were already “primed” by a previous high-conflict trial (as also demonstrated by Egner and Hirsch, 2005). Moreover, in line with our *a priori* hypothesis 1, the Gratton effect was statistically only present with an intact DLPFC (i.e., following sham stimulation), but not after inhibitory brain stimulation of the right prefrontal cortex.

Thus, the DLPFC seems to have been centrally involved in solving the response conflict induced by incongruent Stroop trials, and it did so effectively under “high-control” conditions (i.e., after previous incongruent trials). These findings are in line with prominent models of cognitive control that assume a regulatory function of the DLPFC and an additional role of medial prefrontal areas for the monitoring of situations demanding increased executive control, specifically in terms of high response conflict (Botvinick et al., 2001; Botvinick et al., 2004; Ridderinkhof et al., 2004). In future studies, it could be interesting to directly study the interplay between medial and lateral prefrontal areas in this task context—the medial prefrontal cortex is not well-accessible by fNIRS, but would be by fMRI—and potentially assess effects of inhibitory neurostimulation also for the anterior cingulate cortex (ACC) as a target region (with an appropriate stimulation device and protocol). In this context, it is interesting to note that Lehr et al. (2019) used theta-range tACS (transcranial alternate current stimulation) over the frontal cortex and found a reduced Stroop effect; midfrontal theta is strongly linked to processes of conflict monitoring and error detection in the anterior cingulate cortex (Cohen, 2014; Cohen and Donner, 2013; Ridderinkhof et al., 2004). With respect to alternative interpretations for—and potential confounders of—conflict adaptation effects in certain Stroop or Flanker tasks particularly in terms of repetition priming (Braem et al., 2019; Mayr et al., 2003), we do not believe that this factor had a relevant impact in our case, due to our relatively large stimulus set of a total of 30 different picture/sound combinations (leading to mean stimulus repetitions of less than 5 percent; but see limitations below).

It should briefly be discussed that our current behavioral findings of rTMS effects on Stroop measures are partly in contrast to previous studies showing no effects of excitatory rTMS interventions over the *left* DLPFC on Stroop interference (just overall reduced RTs; Parris et al., 2021; Vanderhasselt et al., 2006; Li et al., 2017). This may be due to different roles of both hemispheres in conflict processing and resolution, with a specific contribution of the right DLPFC for interference control (see also Friehs et al., 2020). This seems to be confirmed by one previous study applying excitatory rTMS over the *right* DLPFC and finding a positive effect on top-down attentional processes specifically in task blocks with a high expectancy of incongruent stimuli (which is somewhat comparable to Stroop trials following previous incongruent trials, thus inverse-mirroring our own findings under high-control conditions).

Considering the neuroimaging results, we found a clear effect of the inhibitory stimulation over relatively broad areas of the prefrontal cortex (see Figure 3), which confirms that the TBS intervention had the intended effect (hypothesis 3a). This broad prefrontal effect, which exceeded the focal stimulation site, is in line with both EEG and fMRI data showing generalized effects on contra-hemispheric activity and network connectivity following different rTMS manipulations, amongst others within the cognitive control network (Gratton et al., 2013; Ilmoniemi et al., 1997; Komssi et al., 2002). Furthermore, directly contrasting activation patterns for conditions underlying the Gratton effect (i.e., incongruent following incongruent vs. incongruent following congruent trials) revealed a cluster of channels within the right DLPFC and adjacent OFC that was more strongly activated in the critical “high conflict–high control” condition. Thus, this area of the right prefrontal cortex may have been specifically related to adjustments in cognitive control elicited by preceding conflictual trials, which is in line with our *a priori* hypothesis 3b and also confirms our choice to stimulate the right DLPFC to test for a causal involvement of just this region in the resolution of response conflict.

In the auditory cortex, lower activation was observed specifically for visual targets (where auditory input served as a distractor) on conflictual (i.e., incongruent) trials when the previous trial had also been incongruent so that cognitive control should have already been established. Interestingly, this effect disappeared following inhibitory stimulation of the right DLPFC and was significantly correlated with (remaining) DLPFC activation (in terms of negative correlations between auditory cortex and DLPFC activation in the “high conflict–high control” condition after verum stimulation, but only at an uncorrected significance level). This pattern of findings suggests that suppression of auditory processing on incongruent visual targets (i.e., downregulation of distracting sensory input) may in fact be one of the mechanisms used by the cognitive control system to manage high sensory conflict (as similarly postulated for the visual system; Kastner and Ungerleider, 2000). The fact that our auditory findings were right-lateralized is furthermore in line with findings of previous studies reporting right-hemispheric dominance in auditory cortex responses to speech sounds based on both EEG source localization (Dimitrijevic et al., 2013) and combined EEG/fNIRS (Steinmetzger et al., 2020).

Regarding the second task-relevant sensory region of interest, the face processing component (N170; putatively reflecting FFA activity) showed a stronger conflict-related decrease in amplitudes for auditory targets following sham compared to verum stimulation. In other words, processing of distracting visual input was more markedly suppressed with an intact DLPFC, indicating a specific top-down influence of the cognitive control system on a face-specific sensory area (FFA) which can be disrupted through inhibitory rTMS of the right DLPFC. These findings are generally in line with previous studies suggesting neural mechanisms of distractor suppression in both the visual (Munneke et al., 2011; Polk et al., 2008; Ruff and Driver, 2006) and auditory system (Stewart et al., 2017). In contrast, we found no indication of a target-feature amplification previously reported for the visual system in classical Stroop experiments (Polk et al., 2008; Purmann and Pollmann, 2015; see also Egner and Hirsch, 2005). This may be due to general differences in the current paradigm, that was based on a multisensory (audio-visual) Stroop task, which may have evoked a different set of mechanisms as compared to studies restricted to the visual domain.

Some limitations of this study should be considered for the interpretation of the results and future projects. First and foremost, out of a total of *N* = 31 participants only different sub-samples could be included in the behavioral (*n* = 22), fNIRS (*n* = 21) and EEG analyses (*n* = 25), so not the same individuals contributed equally to each of these domains, and correlations between fNIRS and EEG (or fNIRS/EEG and behavioral) data did not include the complete study sample. Even though sample size calculations performed prior to this study indicated a minimum required sample size of *N* = 19 (and we aimed to include at least 20 participants in our final analyses, which we did), this limitation should be considered in the interpretation of our findings.

From a more conceptual standpoint, in future studies, other brain areas which are known to be involved in the Stroop task (e.g., the ACC; Bench et al., 1993) should also be measured, as they might compensate for the reduced activity of the DLPFC after verum stimulation and could bring new insights into the functional architecture of cognitive control. Additionally, a second control condition applying verum stimulation outside the cognitive control network (i.e., inhibitory stimulation of another cortical area) would have sharpened the specific interpretation of our data and reduced the possibility that differences in the subjective experience of sham and verum stimulation may have contributed to our results.^3^ Please note that we tried to prevent just that by using a specific placebo-verum stimulation coil, which stimulates the scalp by smooth electrical impulses also in the sham condition (see Methods section). In this same context, post-hoc questionnaires revealed that 23 out of 31 participants indicated that they had noticed some differences between rTMS protocols (verum vs. sham); however, despite this apparent prominence of subjectively perceived differences, only 54.8% of the complete sample correctly guessed for which of the two sessions they had received “real” stimulation, which is—surprisingly—just slightly above chance-level.

Regarding our use of the N170 as an electrophysiological marker of FFA activation, it should be noted that—while the fusiform gyri are among the regions most consistently reported as the neuroanatomical source of the N170 effect (e.g., Itier and Taylor, 2002; Rossion et al., 2003)—other regions have been suggested to contribute to this ERP (e.g., Shibata et al., 2002) and the small number of EEG electrodes used in the current study prevents us from conducting a source analysis. Regarding our second sensory target area, it could be argued that the auditory cortex is not the only possible task-related region of interest, given that interference may be modulated at the semantic (instead of the sensory) level. Therefore, effects might be observed in more anterior regions of the temporal cortex rather than the auditory cortex itself; however, due to the relatively low spatial resolution of fNIRS—combined with the fact that we used a functional channel of interest analysis relying on the most strongly activated NIRS channel per individual—it is not impossible that these more anterior regions contributed to our data anyway. In this context, it should also be mentioned that—in future studies—one possibility would be to include a localizer task in the design, in order to identify critical (sensory) regions on a participant-by-participant basis (see Polk et al., 2008). It should also be kept in mind that—in order to optimize the Stroop effect—different SOAs were employed for the two modalities, hampering any direct comparisons between the visual and auditory task instruction.

Another potential limitation of our design concerns the inclusion of a relatively low number of stimulus categories and corresponding responses, so that some priming effects due to partial stimulus repetitions cannot be excluded (Puccioni and Vallesi, 2012). And finally, the effects of TMS on the DLPFC are controversial (Grossheinrich et al., 2009; Woźniak-Kwaśniewska et al., 2014); however, beside our control of the (intended inhibitory) effects via fNIRS, additional findings in the literature also confirm the reproducibility of TMS effects over brain areas other than the motor cortex (e.g., Chung et al., 2018; Tupak et al., 2013). Nevertheless, the actual effect of a TMS treatment should be observed and discussed carefully.

Taken together, our combined fNIRS/EEG data indicate that—with an intact right DLPFC—the cognitive control system invokes a downregulation (i.e., suppression) of distracting sensory input on high-conflict trials in both visual (FFA) and auditory cortices. After an inhibitory stimulation of the right DLPFC, these effects were significantly reduced—and a behavioral Gratton effect no longer present—indicating a central involvement of the DLPFC in the top-down control of task-relevant sensory areas and the resolution of Stroop-induced response conflict. In contrast to Egner and Hirsch (2005)—who reported an *amplification* of task-relevant stimulus processing in the FFA on high-conflict trials for a unimodal (purely visual) Stroop task—we found indications of distractor *suppression* on high-conflict trials within both visual and auditory regions of interest for our multisensory Stroop task.

Zooming out from this very specific neuroscientific perspective, the Stroop task more generally speaking is a complex cognitive task involving mechanisms of selective attention, perception, problem solving, working memory, response inhibition, and conflict resolution—processes that are central to the field of cognitive psychology. Shedding some light on the neural basis of some of these processes provides the groundwork for developing targeted interventions for clinical groups with impairments in filtering out task-irrelevant information and/or resolving response conflict. It is also likely that the conclusions drawn from our data are not specific to the Stroop task, but reflect more general mechanisms of top-down control in light of competing response options. In a world where the information processing system is constantly flooded by diverse environmental stimuli that often cue contradictory actions (e.g., different traffic signals for bike vs. car lanes), focusing our attention on behaviorally relevant aspects of our surroundings is a key skill with broad theoretical and practical relevance. Significant correlations of our Stroop measures with different questionnaire data assessing attentional control and cognitive failures confirm the broader relevance of our findings.

## 5 Conclusion

Based on the current study, it can be concluded that the DLPFC is critically involved in the top-down control of sensory areas in high-conflict situations. Previously, both stimulus amplification and inhibition have been suggested as relevant mechanisms employed by the cognitive control system (Chawla et al., 1999; Kastner and Ungerleider, 2000; Treue and Maunsell, 1996). Here, we found only indications of a distractor suppression in task-relevant sensory areas of both modalities (FFA and auditory cortex, respectively). This conflict-related modulation of sensory cortices was significantly reduced following temporary inhibition of the prefrontal cortex, suggesting that the DLPFC invoked specialized response patterns in the FFA and auditory cortex to optimize behavioral responses in light of competing response options.

The results of this study provide new insights into the neural mechanisms used by the DLPFC to exert top-down control over different sensory areas in high-conflict situations. In the current multimodal (audiovisual) Stroop task, an inhibition of distracting sensory input was identified as the primary mechanism invoked to reduce response conflict. Taken together, our combined fNIRS/EEG and behavioral data provide interesting insights into the specific implementation of cognitive control by the prefrontal cortex under conditions of high sensory and response conflict.

## Data Availability

The raw data supporting the conclusions of this article will be made available by the authors, without undue reservation.

## Footnotes

^1^Please note that this multisensory task differs conceptually from paradigms underlying the extensive body of literature on multisensory (and particularly audio-visual) conflict and integration, such as the ventriloquist illusion, the McGurk effect or auditory driving. The mechanisms underlying the resolution of multisensory conflict and integration in these basic sensory contexts have been well studied (e.g., Roach et al., 2006; Rohe et al., 2019), particularly for cross-modal features that are linked by being attributed to a specific object (Diaconescu et al., 2011). However, in the current paradigm, audio-visual information cannot be integrated into a single percept nor linked or attributed to a specific person. Instead, separate information is presented through visual and auditory channels, consistently requiring attentional focus on one of these sensory channels to detect the identity of the presented celebrity, whether auditory or visual. In this paradigm, the visually presented person does not speak, and the speaker’s voice is unrelated to the depicted person. Additionally, the names read out never match the identity of the picture, just sometimes the response category.

^2^Please note that the opposite was true for current congruent (i.e., low-conflict) trials: Here, IES were significantly lower following congruent (*M* = 620.81, *SD* = 118.55) as compared to incongruent trials (*M* = 682.34, *SD* = 121.69; *t*_21_ = 4.30, *p* < 0.001; direct comparison of the “Gratton difference measure” between current congruent vs. current incongruent trials: *t*_21_ = 5.28, *p* < 0.001).

^3^However, use of the contralateral DLPFC as a “control region” would probably not be feasible due to the controversy still surrounding the relative contribution of the left and right DLPFC to interference resolution/cognitive control (see above), even though this is common practice for other tasks and cognitive functions (e.g., Vallesi et al., 2007).
