# Supplementary material for: The DLPFC is centrally involved in resolving Stroop conflicts, suppressing distracting sensory input within the auditory and visual system
Source: Front Psychol. 2024 Oct 18;15:1427455. doi: 10.3389/fpsyg.2024.1427455 (PMC11528708; doi:10.3389/fpsyg.2024.1427455)
Supplement: Supplementary file 1 [file Table_1.DOCX]

Supplementary Material

The DLPFC is causally involved in resolving Stroop conflicts, with different attentional mechanisms within the auditory and visual system

Ann-Christine Ehlis*, Lisa Zarantonello, Florian B. Haeussinger, Tim Rohe,
David Rosenbaum, Andreas J. Fallgatter, Moritz J. Maier

*** Correspondence:** Ann-Christine Ehlis: ann-christine.ehlis@med.uni-tuebingen.de

**Pre-study (Determination of optimal SOAs for the audio-visual Stroop task)**

In order to determine the optimal stimulus onset asynchronies (SOAs) for the audio-visual Stroop task, an independent group of 19 participants completed the task with three different SOAs between the target stimulus and the distractor (0 ms, 150 ms and 300 ms), separately for the two target conditions (auditory vs. visual; distractor always simultaneously with or prior to the target). To evaluate the findings, we regarded Stroop effects (i.e., difference measures “incongruent – congruent trials”) regarding both reaction times (RTs) and error rates.

For the *visual task condition*, small but positive Stroop effects occurred for the RTs with SOAs of 150 and 300 ms, while simultaneous presentation of both stimuli resulted in a small but negative Stroop effect. The only significant difference between SOA conditions was observed for the comparison of SOA 0 and SOA 150 (*t*_18_=2.97, *p*<.01, *d*=0.68) with a larger Stroop effect when the auditory distractor was presented 150 ms prior to the picture than when both stimuli were presented simultaneously (see Figure S1). In contrast, SOA did not significantly impact the percentage of errors (difference between congruent and incongruent trials, i.e., error-based Stroop effect) made during the visual task condition (all *p*>.1 for two-sided testing; however, for one-sided testing, SOAs of 150 and 300 ms tended to induce increased error-based Stroop effects as compared to the SOA of 0 ms: *t*_18_=1.523 and 1.672, *p*=.073 and .056, *d*=0.349 and 0.384, respectively; see Figure S2). Based on all of these findings, an SOA of 150 ms was chosen for visual targets of the main study (auditory distractor first).

For the *auditory task condition*, no significant SOA differences occurred for the RT-based Stroop effects (all *t*_18_<1.25, all *p*>.2, all *d*<0.3). However, the Stroop effect reflected by the error rates differed significantly between SOAs, with the strongest effect for an SOA of 0 ms (i.e., with a simultaneous presentation of the two stimuli; difference between error rates for incongruent – congruent trials: 10.96±11.48%) as compared to SOAs of 150 (7.61±12.51%; *t*_18_=2.09, *p*=.05, *d*=0.48) and 300 ms (1.65±8.03%; *t*_18_=4.77, *p*<.001, *d*=1.09; see Figure S2). Therefore, for auditory targets, an SOA of 0 ms was chosen for the main study (i.e., simultaneous presentation of both stimuli).

**

**

**Supplementary Figure 1.** Stroop effect based on the reaction times (RT in ms) in incongruent (I) minus congruent (C) trials for auditory (blue line) vs. visual (red line) targets. For visual targets, the Stroop effect was significantly larger for an SOA of 150 as compared to 0 ms (**p*<.01).

*

***

**Supplementary Figure 2.** Stroop effect based on the error rates (E) in incongruent (I) minus congruent (C) trials for auditory (blue line) vs. visual (red line) targets. The Stroop effect was increased for auditory targets with an SOA of 0 as compared to both 150 (**p*=.05) and 300 ms (****p*<.001).
